# Supplementary material for: Zero-dimensional halide hybrid bulk glass exhibiting reversible photochromic ultralong phosphorescence
Source: Nat Commun. 2024 Jun 29;15:5519. doi: 10.1038/s41467-024-49886-7 (PMC11217438; doi:10.1038/s41467-024-49886-7)
Supplement: Supplementary file 1 — Supplementary Information [file 41467_2024_49886_MOESM1_ESM.pdf]

Supplementary Information

Zero-dimensional Halide Hybrid Bulk Glass Exhibiting Reversible Photochromic Ultralong Phosphorescence

Fei Nie[1], and Dongpeng Yan[1]*

[1]Beijing Key Laboratory of Energy Conversion and Storage Materials, and Key Laboratory of Radiopharmaceuticals, Ministry of Education, College of Chemistry, Beijing Normal University, Beijing 100875, P. R. China (P. R. China)

Correspondence and requests for materials should be addressed to D. Y. (email: yandp@bnu.edu.cn).

Supplementary Figures and Tables

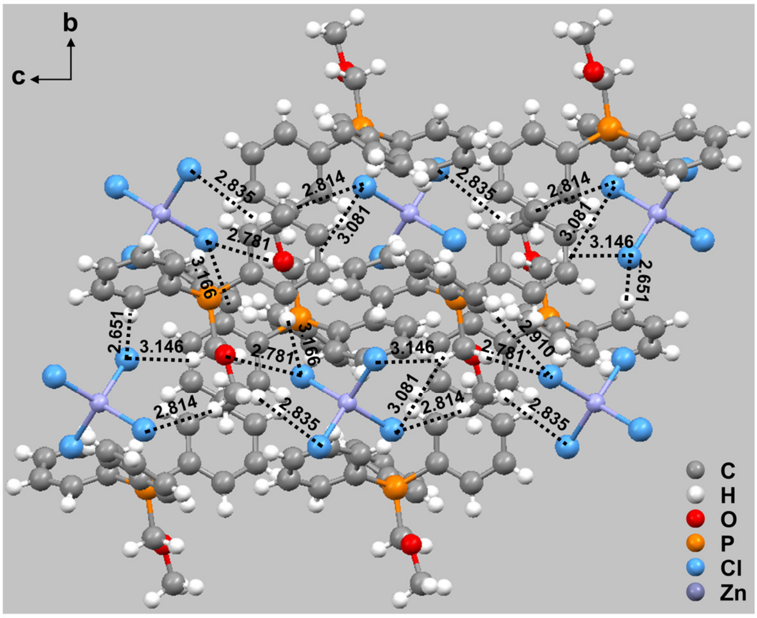

Supplementary Figure 1. The crystal structure of P-Zn as viewed from the a-axis (unit: Å).

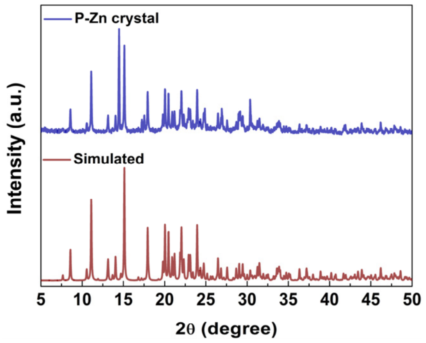

Supplementary Figure 2. The PXRD pattern of P-Zn crystal and the simulated crystal structure data. a.u., arbitrary units.

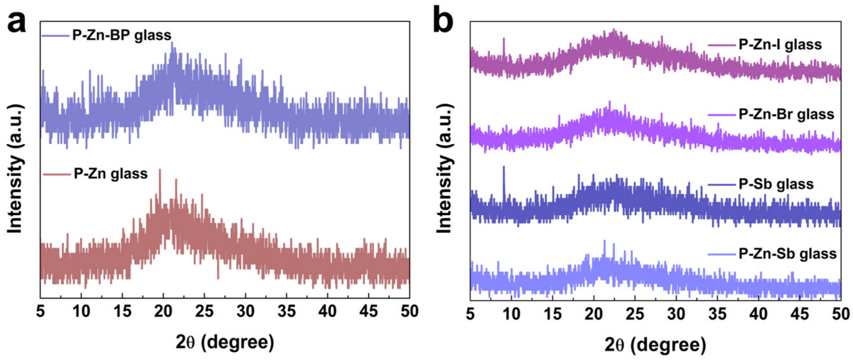

Supplementary Figure 3. (a) The PXRD patterns of P-Zn, P-Zn-BP, (b) P-Zn-Sb, P-Sb, P-Zn-Br, and P-Zn-I glasses. a.u., arbitrary units.

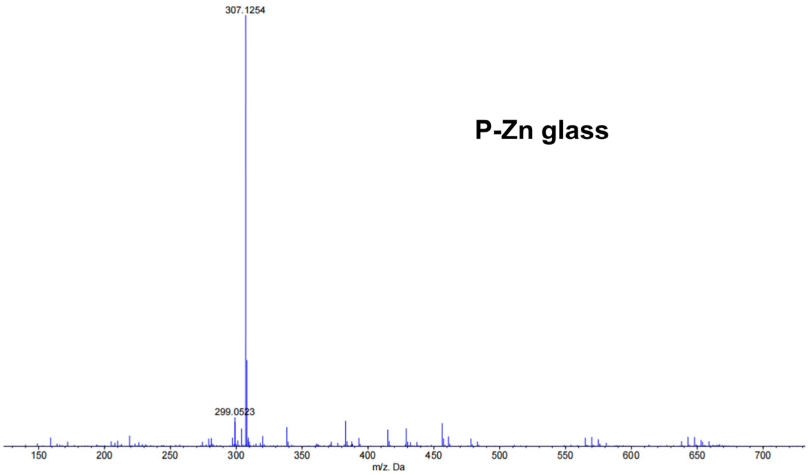

Supplementary Figure 4. The HR-ESI-MS spectrum of P-Zn glass.

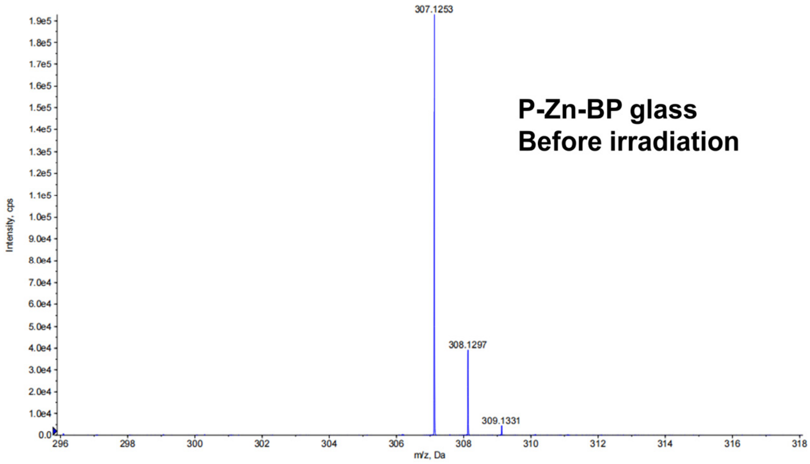

Supplementary Figure 5. The HR-ESI-MS spectrum of P-Zn-BP glass before irradiation.

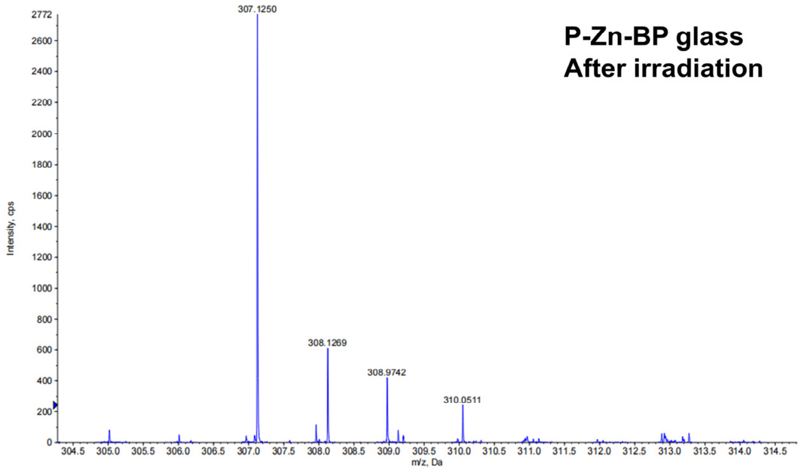

Supplementary Figure 6. The HR-ESI-MS spectrum of P-Zn-BP glass after irradiation.

Supplementary Note 1: The FT-IR results for P-Cl powder, P-Zn crystal, and P-Zn glass

The very close FT-IR spectra between P-Cl powder, P-Zn crystal, and P-Zn glass imply that the main organic component in the crystalline and glassy samples is P^+ cations (Supplementary Fig. 7, 8).

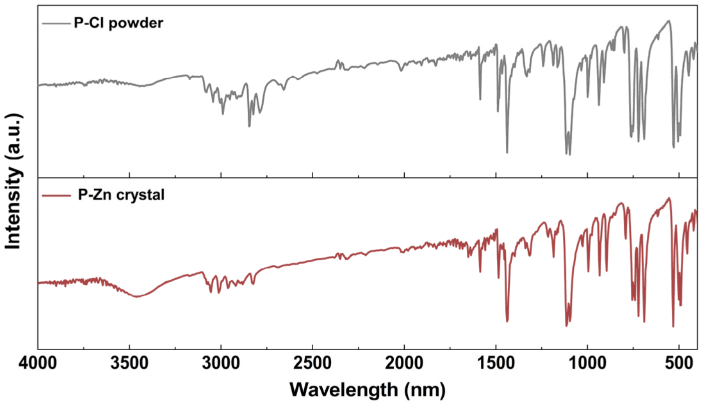

Supplementary Figure 7. The FT-IR results for P-CI powder and P-Zn crystal. a.u., arbitrary units.

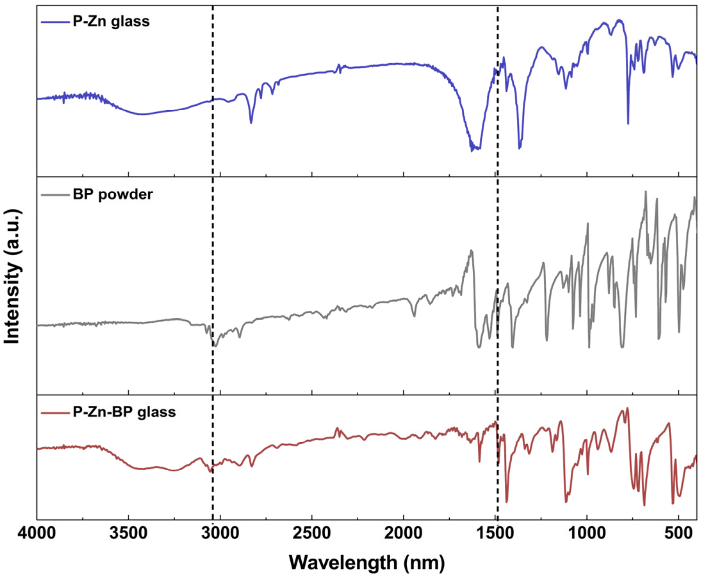

Supplementary Figure 8. The FT-IR results for BP powder, P-Zn and P-Zn-BP glasses. a.u., arbitrary units.

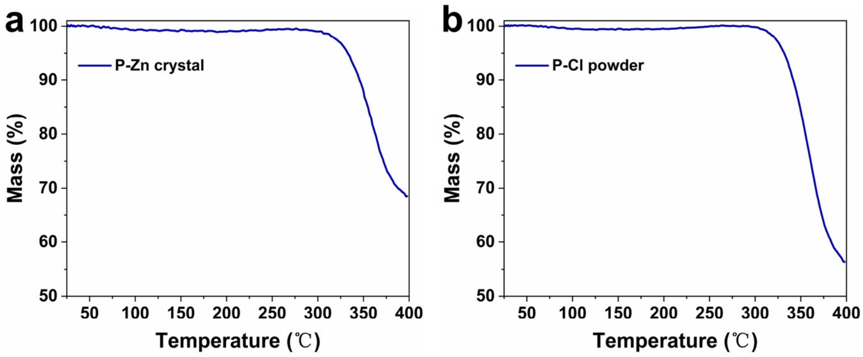

Supplementary Figure 9. (a) The TGA results for P-Zn crystal and (b) P-Cl powder.

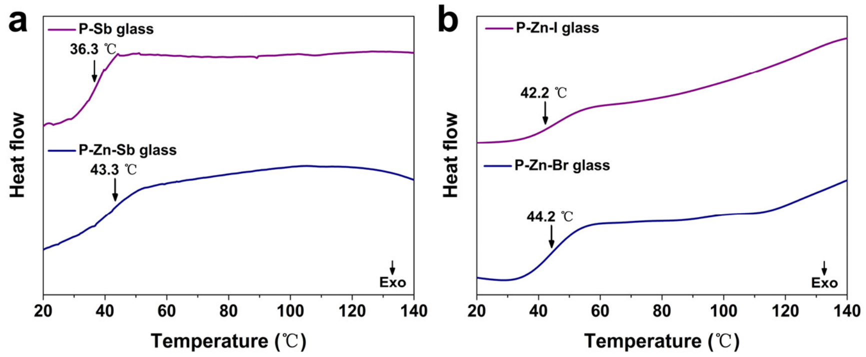

Supplementary Figure 10. The DSC curves of (a) P-Zn-Sb, P-Sb, (b) P-Zn-Br, and P-Zn-I glasses.

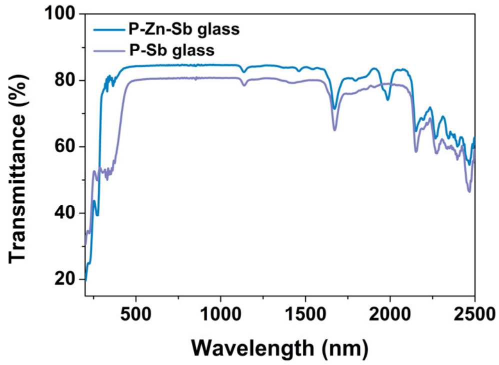

Supplementary Figure 11. UV-Vis-NIR transmittance spectra of P-Zn-Sb and P-Sb glasses.

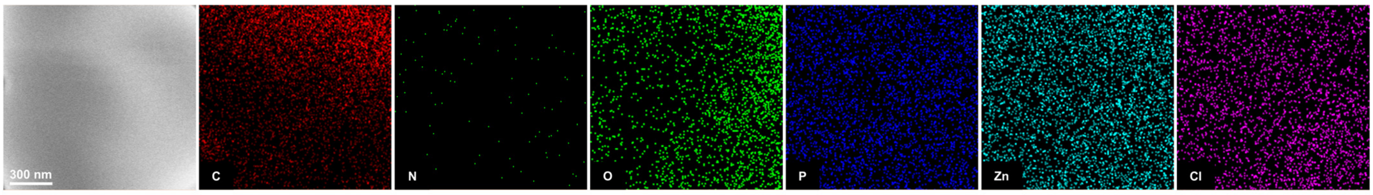

Supplementary Figure 12. The SEM image of P-Zn-BP glass, and its EDS mapping showed C, N, O, P, Cl and Zn with a homogeneous distribution of elements.

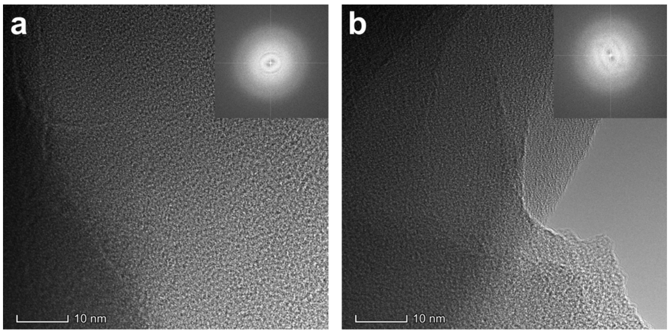

Supplementary Figure 13. High-resolution TEM images and SAED patterns (inset) of (a) P-Zn and (b) P-Zn-BP glasses.

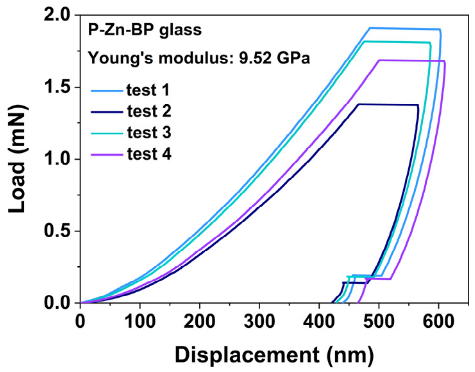

Supplementary Figure 14. The nanoindentation tests of P-Zn-BP glass with the loading-unloading curves. The curves were obtained by conducting measurements at four different points.

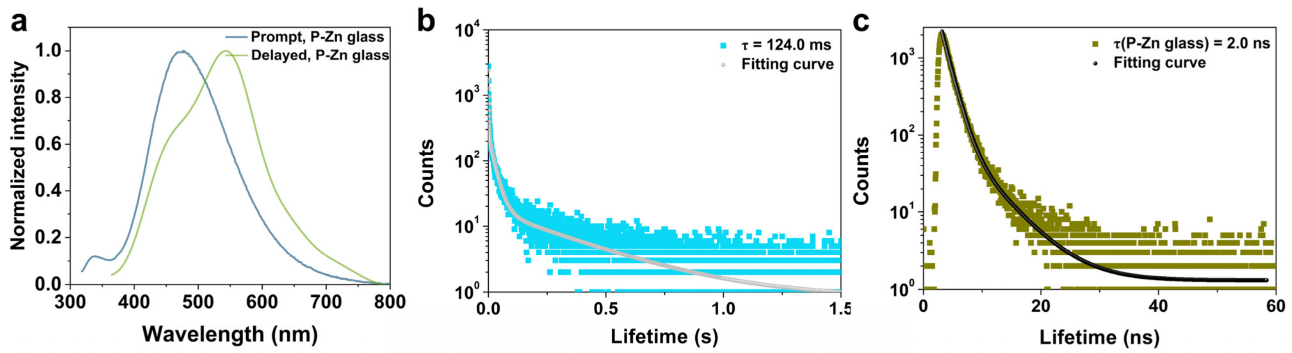

Supplementary Figure 15. (a) Prompt and delayed PL spectra, and (b) phosphorescence decay profile (measured at R.T.) of P-Zn glass prepared by melting of the mixture of P-Cl and $ZnCl_2$. (c) The fluorescence decay profile of P-Zn glass.

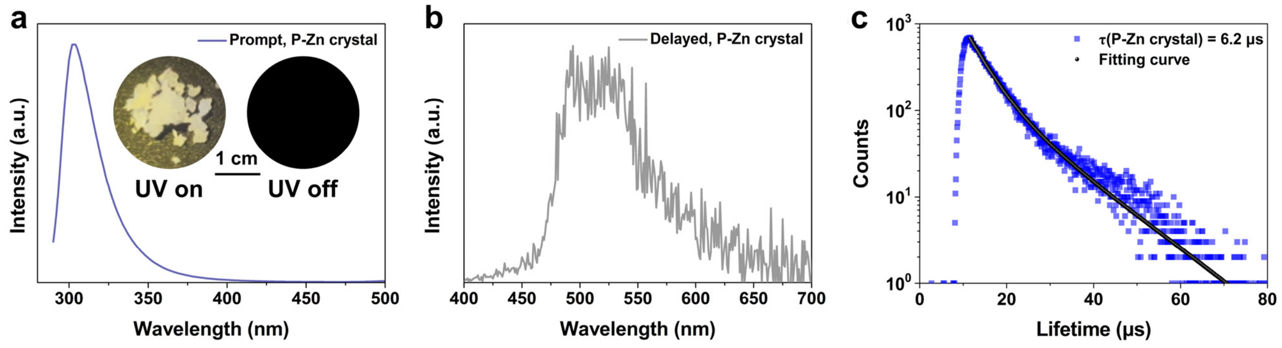

Supplementary Figure 16. (a) Prompt and (b) delayed PL spectra, and (c) phosphorescence decay profile of P-Zn-I glass at 525 nm at R.T.. Inset: the photographs of P-Zn crystal before and after the 365 nm lamp removal. a.u., arbitrary units.

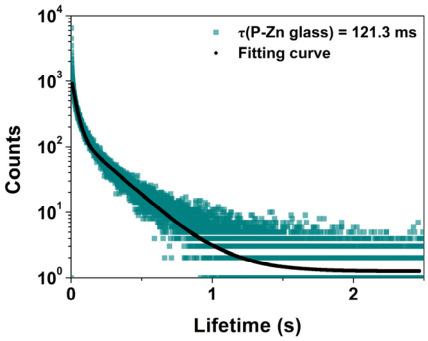

Supplementary Figure 17. Phosphorescence decay profile (at 525 nm, R.T.) of P-Zn glass prepared by melting of P-Zn crystal.

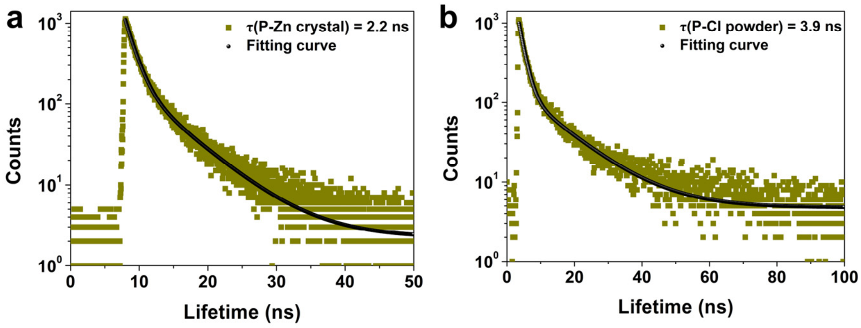

Supplementary Figure 18. The fluorescence decay profiles of (a) P-Zn crystal and (b) P-Cl powder.

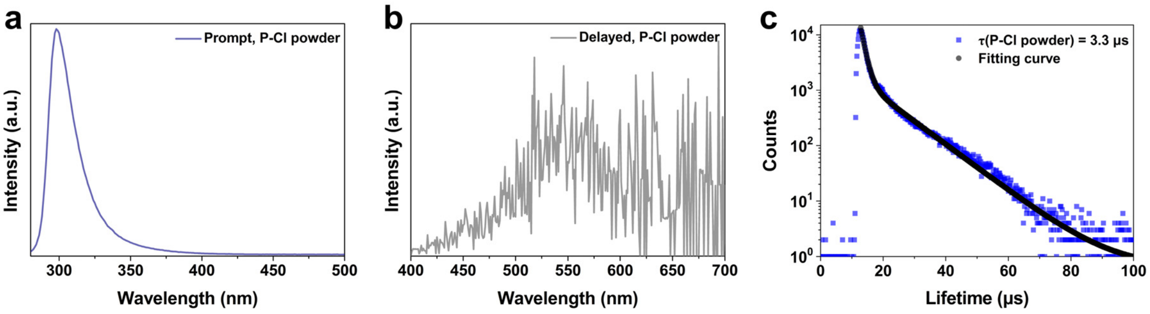

Supplementary Figure 19. (a) Prompt and (b) delayed PL spectra, and (c) phosphorescence decay profile of P-Cl powder at 550 nm at R.T.. a.u., arbitrary units.

Supplementary Note 2: The prompt PL spectrum of solid $ZnCl_2$

The prompt PL spectrum of solid-state $ZnCl_2$ indicates the absence of any photoluminescent properties (Supplementary Fig. 20).

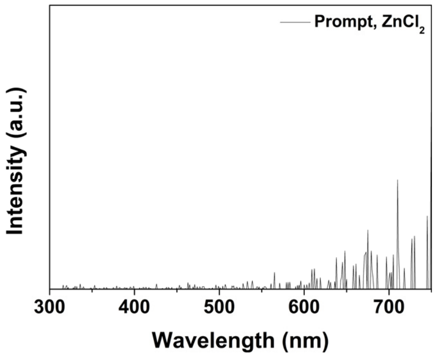

Supplementary Figure 20. Prompt PL spectrum of $ZnCl_2$ powder. a.u., arbitrary units.

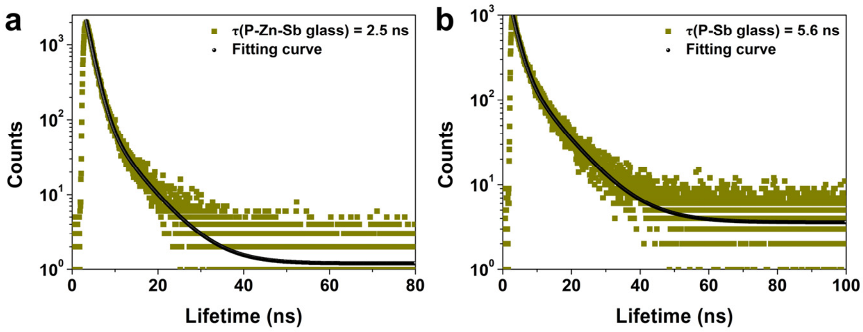

Supplementary Figure 21. (a) The fluorescence decay profiles of P-Zn-Sb and (b) P-Sb glasses.

Supplementary Note 3: The luminous behaviors of P-Zn-Sb and P-Sb glasses

The PL tests reveal that the P-Zn-Sb glass exhibits NIR emissions with two main peaks at 500 and 650 nm in the prompt mode, and phosphorescence emission at 525 nm with the RTP lifetime of 10.3 ms (Supplementary Fig. 22). The P-Sb glass also shows NIR emission at 650 nm in the prompt mode, but weak RTP emission with a microsecond lifetime (4.9 μs) (Supplementary Fig. 23). Thus, it is speculated that the NIR emission of P-Zn-Sb glass is originated from Sb-based OIMHs, and the long-lived RTP could be attributed to presence of Zn-based hybrids in the glassy system.

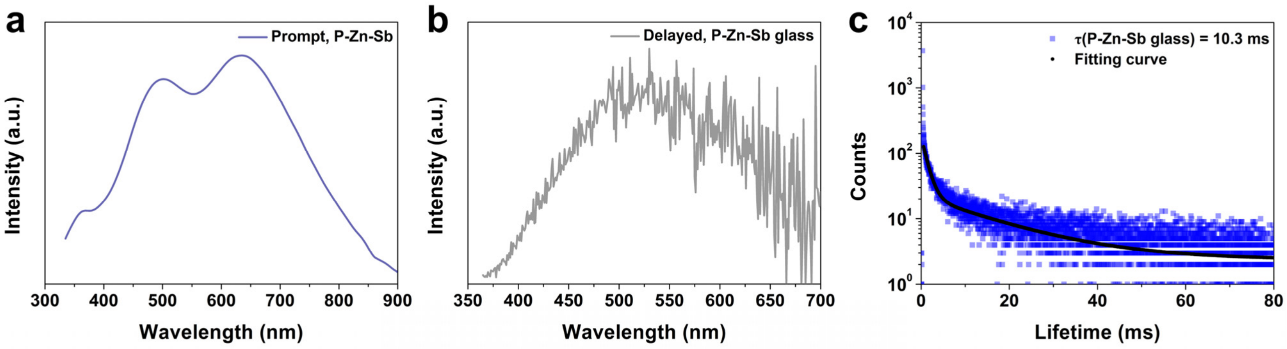

Supplementary Figure 22. (a) Prompt and (b) delayed PL spectra, and (c) phosphorescence decay profile of P-Zn-Sb glass at 525 nm at R.T.. a.u., arbitrary units.

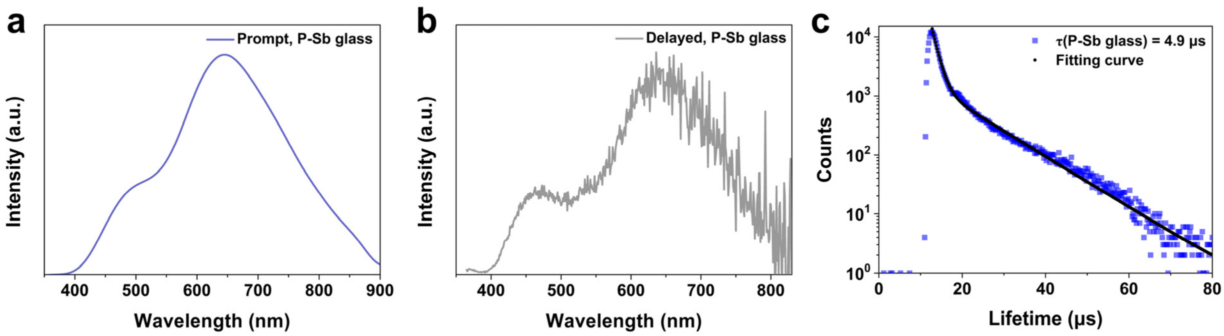

Supplementary Figure 23. (a) Prompt and (b) delayed PL spectra, and (c) phosphorescence decay profile of P-Sb glass at 650 nm at R.T.. a.u., arbitrary units.

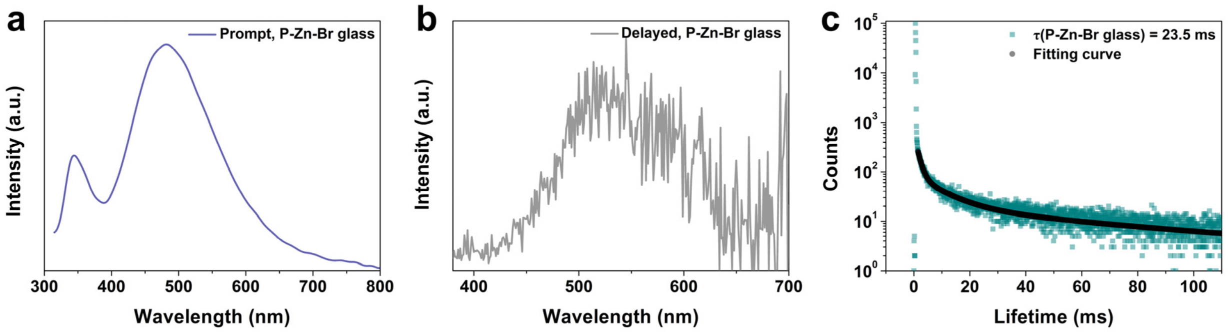

Supplementary Figure 24. (a) Prompt and (b) delayed PL spectra, and (c) phosphorescence decay profile of P-Zn-Br glass at 510 nm at R.T.. a.u., arbitrary units.

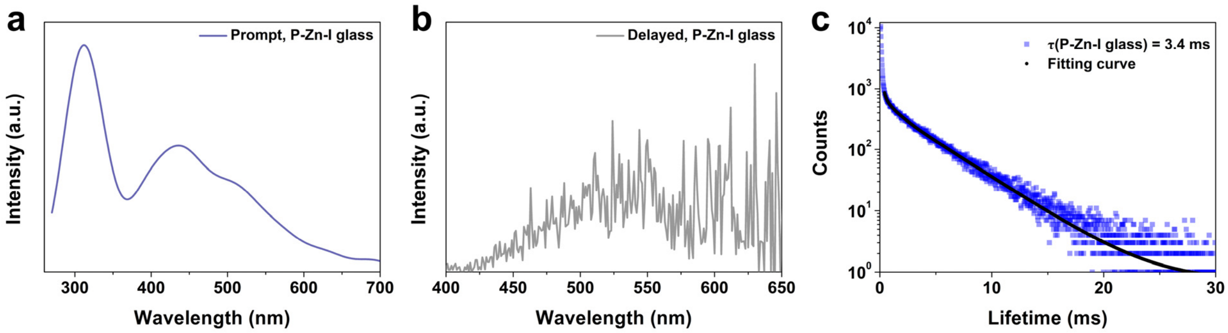

Supplementary Figure 25. (a) Prompt and (b) delayed PL spectra, and (c) phosphorescence decay profile of P-Zn-I glass at 525 nm at R.T.. a.u., arbitrary units.

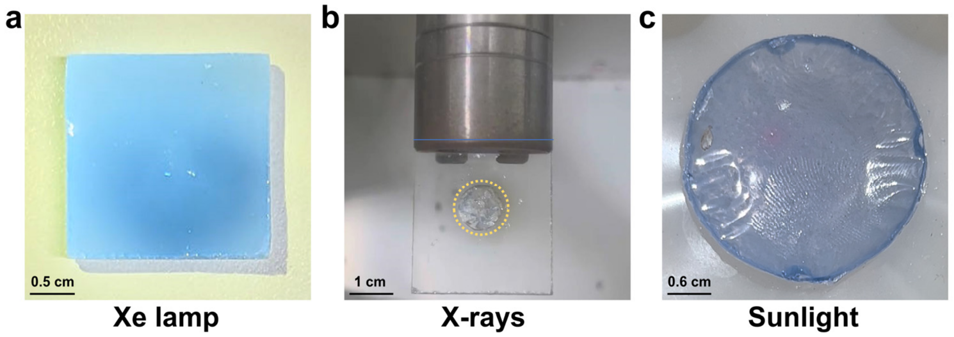

Supplementary Figure 26. The photographs of P-Zn-BP glass after the irradiation by (a) Xe lamp, (b) X-rays and (c) sunlight.

Supplementary Note 4: The luminous behaviors of colorless P-Zn-BP glass

The investigation into the predominant emission band at 467 nm in the prompt PL spectrum of P-Zn-BP glass before irradiation has been conducted (Supplementary Fig. 27a). Time-resolved PL decay curves of the glass reveal a 2.0 ns lifetime at 350 nm (Supplementary Fig. 27b) and a longer lifetime of 107.9 ms at 525 nm (Fig. 3e). Additionally, temperature-dependent prompt and delayed PL spectra exhibit a systematic decrease in intensity with rising temperature from 77 to 297 K (Supplementary Fig. 28). Notably, a significant overlap is observed between the emission bands in the prompt and delayed PL spectra (Fig. 3c, 3d). Importantly, two emission bands at 350 and 467 nm appear for the glass after coloration, possibly due to the phosphorescence intensity of the glass decreasing faster than the fluorescence intensity after illumination. These findings collectively indicate that the emissive band in the prompt PL spectrum of colorless P-Zn-BP glass stems from a combination of fluorescence and phosphorescence, a phenomenon commonly observed in many RTP materials[1–3].

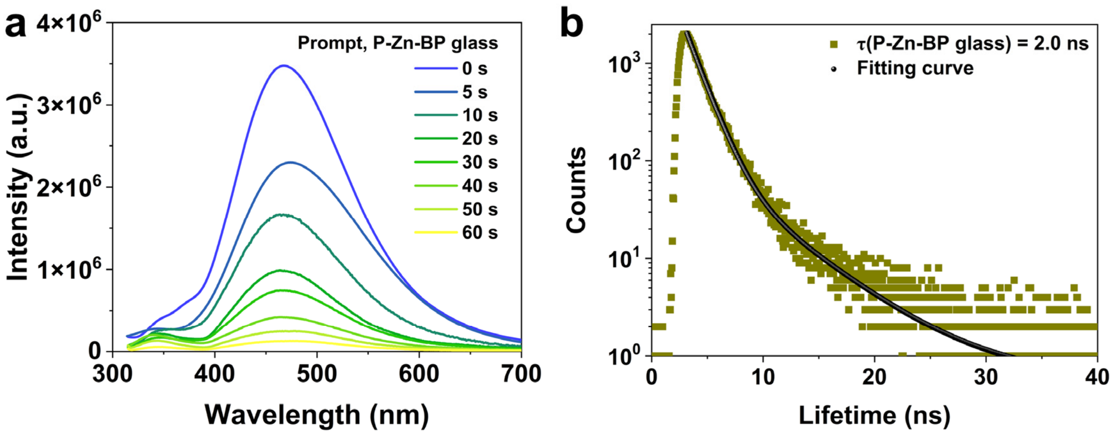

Supplementary Figure 27. (a) Prompt PL spectra P-Zn-BP glass at different irradiation times. (b) The fluorescence decay profile of P-Zn-BP glass at 350 nm. a.u., arbitrary units.

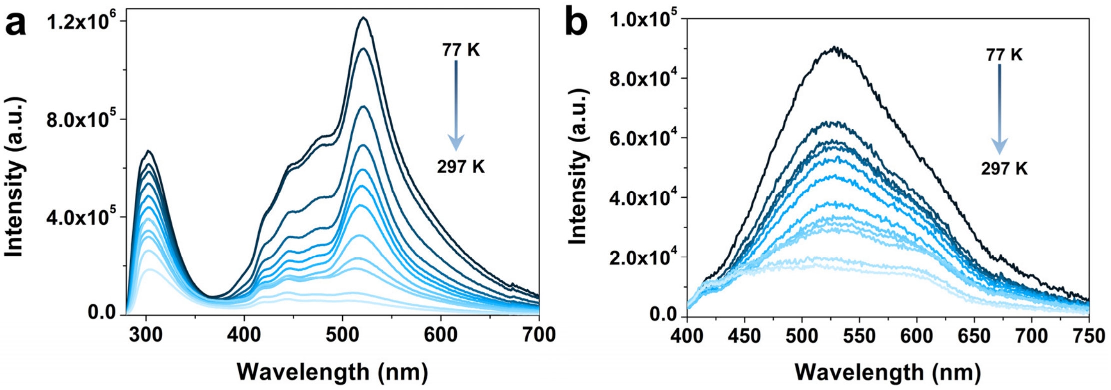

Supplementary Figure 28. (a) Prompt and (b) delayed PL spectra of P-Zn-BP glass from 77 to 297 K. a.u., arbitrary units.

Supplementary Note 5: The RTP generation for P-Zn-BP glass before and after coloration

The phosphorescence observed in BP-doped glass before irradiation mainly stems from the organic component, as evidenced by its RTP emission and excitation spectra resembling those of P-Zn glass (Fig. 3d, Supplementary Fig. 15a, 29). Differently, the RTP emission observed in P-Zn-BP glass after coloration seems to arise from radiative transitions of P^+ cations with the energy level of BP radicals as a mediator[4, 5]. This is supported by the reduced intensity of phosphorescent emission and excitation, along with a slight blue-shift (from 523 to 514 nm) in the RTP emission following photoirradiation (Fig. 3d, Supplementary Fig. 29).

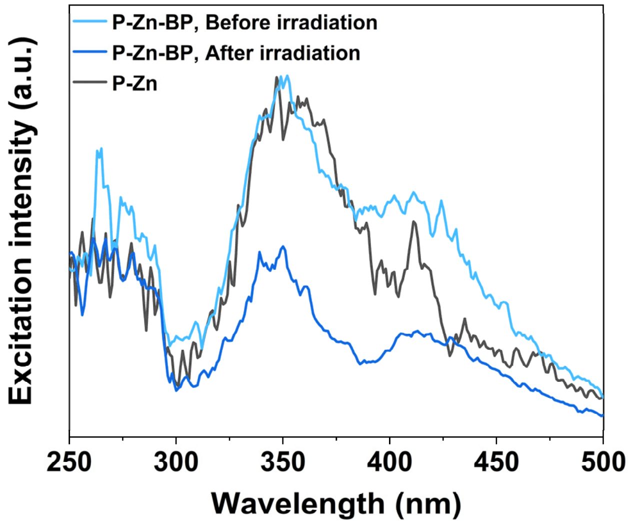

Supplementary Figure 29. Delayed PL excitation spectra of P-Zn glass and P-Zn-BP glass before and after photoirradiation. a.u., arbitrary units.

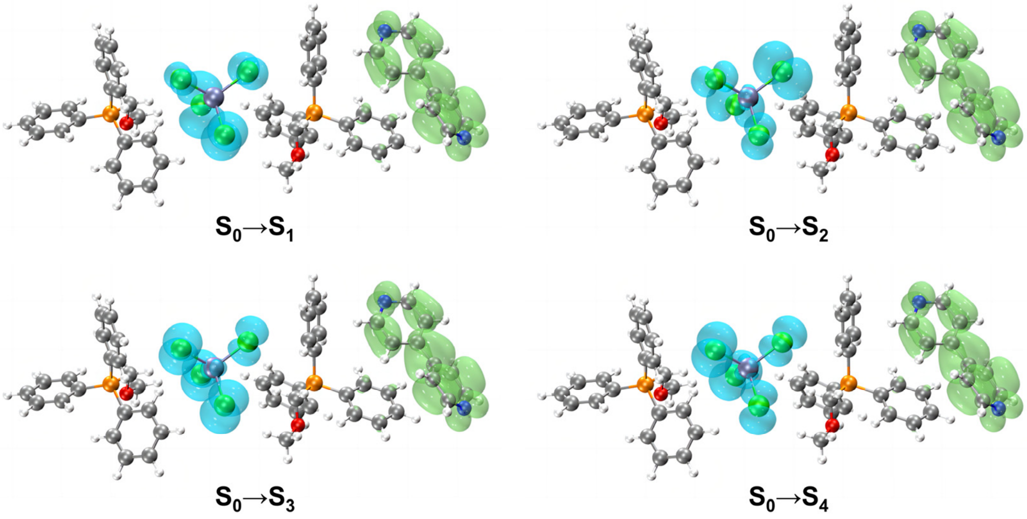

Supplementary Figure 30. Distribution maps of the hole and electron of $S_0 \rightarrow S_n$ transition. Green represents the electron-accept part, and the blue means the hole.

Supplementary Note 6: The calculation results of ESP distribution maps for P-Zn OIMHs

In the case of P-Zn OIMHs, the blue regions representing more negative ESP values are predominantly concentrated around the Cl anions, highlighting the robust electron-donating capacity of these Cl anions (Supplementary Fig. 31). This observation aligns with the outcomes from the distribution maps of holes and electrons (Supplementary Fig. 30).

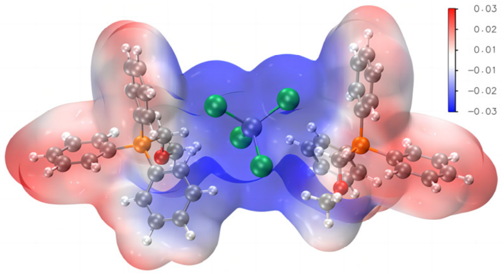

Supplementary Figure 31. Calculated ESP distribution maps of P-Zn. Bluish colors represent more negative ESP values, and reddish colors mean more positive ESP values.

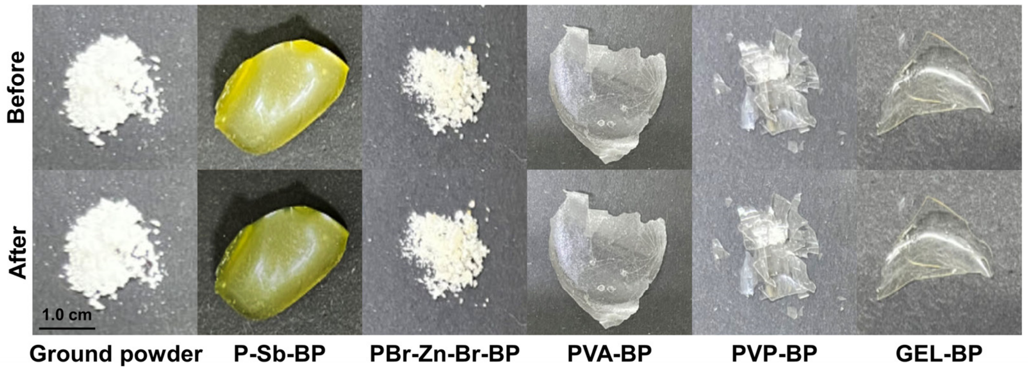

Supplementary Figure 32. The photographs of the ground powder composed of P-Cl, $ZnCl_2$ and BP (in a molar ratio of 2:1:0.02), BP-dope glasses (P-Sb-BP and PBr-Zn-Br-BP) and BP-doped polymeric films (PVA-BP, PVP-BP and GEL-BP) captured before and after exposure to a 365 nm lamp (5 W) for 5 min.

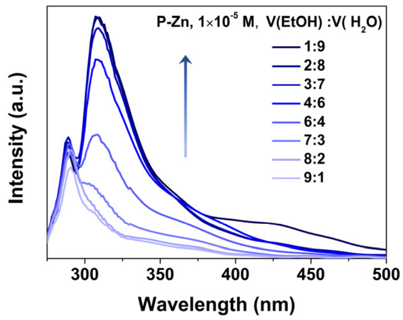

Supplementary Figure 33. The prompt PL spectra of P-Zn in $EtOH$-H_2O mixture (1×10^{-5} M) with different volume ratios of EtOH and H_2O. a.u., arbitrary units.

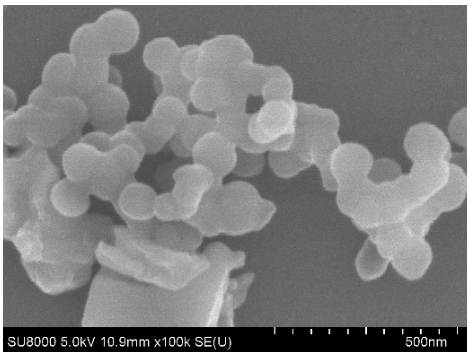

Supplementary Figure 34. The SEM image of the aggregates of P-Zn formed in an EtOH-H_2O mixture with a volume ratio of 9:1 (concentration: 1×10^{-5} M).

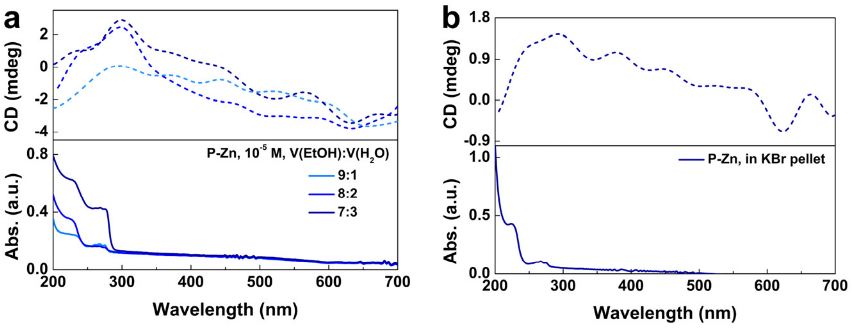

Supplementary Figure 35. (a) The CD spectra of P-Zn in EtOH-H_2O mixture (1×10^{-5} M) with different volume ratios of EtOH and H_2O. (b) The CD spectra of solid-state P-Zn dispersed in KBr pellet. a.u., arbitrary units.

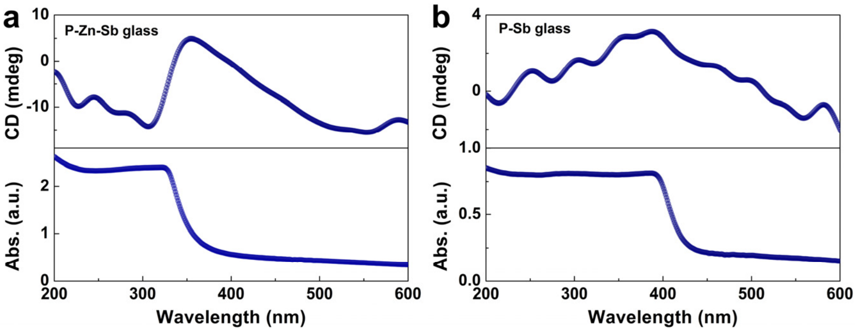

Supplementary Figure 36. The CD absorption spectra of (a) P-Zn-Sb and (b) P-Sb glasses at R.T.. a.u., arbitrary units.

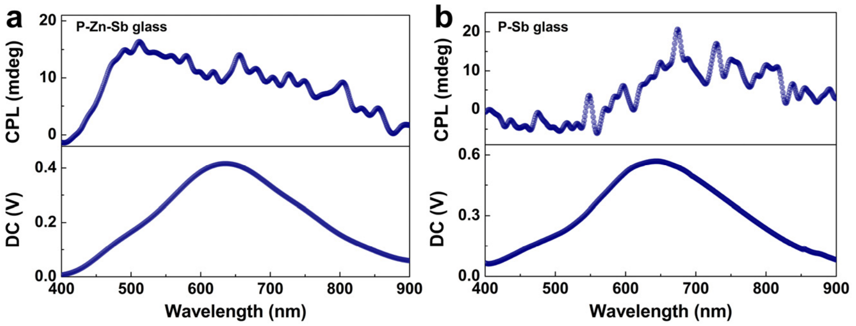

Supplementary Figure 37. The CPL spectra of (a) P-Zn-Sb and (b) P-Sb glasses excited by 320 nm at R.T.. a.u., arbitrary units.

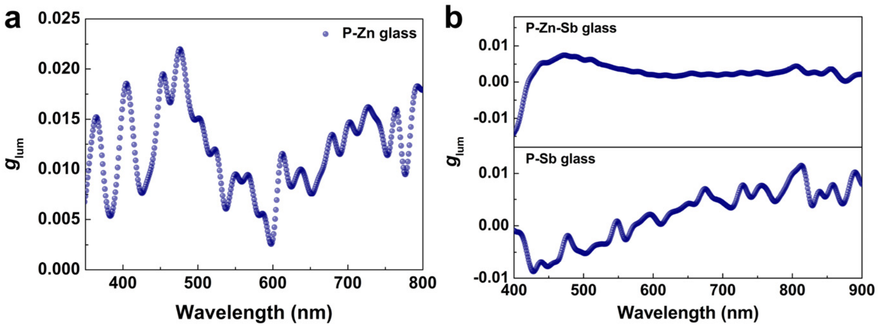

Supplementary Figure 38. The CPL dissymmetry factors of (a) P-Zn and (b) P-Zn-Sb and P-Sb glasses at R.T..

Supplementary Table 1. Recently reported photochromic materials with photocontrollable RTP.

Sample	Component	Photoresponsive time	Recovery time	RTP lifetime	Ref.
Crystal	Complex 1	1.5 min	60 min[a]	99.2 ms	6
	$\{[Zn(bcbpy)_{0.5}(IPA)]•CH_3CN\}_n$	2 min	30 min[a]	100.8 ms	7
	$\{[Zn_2(cbbpy)_2(IPA)_2]•4H_2O\}_n$	1 min	60 min[a]	88.0 ms	8
	$\{[Zn(cbbpy)(HBTC)(H_2O)]•2H_2O\}_n$	4.5 min	30 min[a]	93.1 ms	9
	$[Dy_2(H_2\text{-HEDP})_3(H\text{-HEDP})]$ $•H_3\text{-TPB}•8H_2O$	20 min	240 min[a]	26.2 ms	10
	$[Dy_2(H_2\text{-HEDP})_4]_3•2(H_3\text{-TPA})$ $•2(H_4\text{-HEDP})•x\text{solvent}$	40 min	*	26.6 ms	11
	$(H_4BDMPy\text{-}Br_2NDI)•(NMP)_4•(HPW_{12}O_{40})$	3 min	–	1.1 ms	12
	$(H_4BDMPy\text{-}NDI)•(NMP)_4•(HPW_{12}O_{40})$	0.5 min	A few hours[b]	0.3 ms	13
	$(H_4BDMPy\text{-}NDI)•(NMP)_{12}•(H_3SiW_{12}O_{40})_2$	3 min	A few hours[b]	9.0 ms	13

	$(H_3\text{-TPB})\bullet[Zn_6(H\text{-HEDP})(HEDP)_3(H_2O)_2]\bullet 5H_2O$	60 min	*	0.08 ms	14
	$(H\text{-TPB})\bullet[Zn_3(H\text{-HEDP})(HEDP)(H_2O)]\bullet 2H_2O$	60 min	90 min[a]	0.32 ms	14
	Complex 1	2 min	240 min[b]	40.3 ms	15
	Complex 2	1 min	30 min[a]	66.8 ms	15
	Complex 3	1 min	180 min[b]	31.4 ms	15
**Powder**	Polymer C9	20 s	16 min[b]	14.3 ms	16
**Film**	P1	10 min	8 min[a]	20.2 ms	17
	P2	10 min	8 min[a]	2.1 ms	17
	NDIA/PVA	4 min	–	34.0 ms	18
**Glass**	P-Zn-BP	1 min	3 min[a]	107.9 ms	This work

Note: 1. The photo-response time was defined here as the time it took for the intensities of new absorption bands of the photochromic samples to reach saturation. 2. "–": recover time was not mentioned; "*": the color could not be recovered. 3. [a]: heating; [b]: put in the dark environment.

Supplementary Table 2. Crystal data for P-Zn crystal.

**Sample**	**P-Zn**
**Molecular Formula**	$C_{40}H_{40}O_2P_2Zn$
**Molecular Weight**	821.83
**Density (g cm^{-3})**	1.439
**Crystal system**	Monoclinic
**Space group**	*C2/c*
**a (Å)**	14.2218(3)
**b (Å)**	15.9189(4)
**c (Å)**	17.6229(5)
**α (deg)**	90.00
**β (deg)**	108.090(3)
**γ (deg)**	90.00
**V (Å^3)**	3792.53(18)

**Z**	4
***F* (000)**	1696.0
S	0.992
**R [I > 2σ(I)]**	R_1 = 0.0493
	wR_2 = 0.1342

Supplementary References

1. Ye, W. et al. Confining isolated chromophores for highly efficient blue phosphorescence. *Nat. Mater.* **20,** 1539–1544 (2021).
2. Zhou, B., Xiao, G. and Yan, D. Boosting Wide-Range Tunable Long-Afterglow in 1D Metal–Organic Halide Micro/Nanocrystals for Space/Time-Resolved Information Photonics. *Adv. Mater.* **33,** 2007571 (2021).
3. Zhou, B. and Yan, D. Color-tunable persistent luminescence in 1D zinc–organic halide microcrystals for single-component white light and temperature-gating optical waveguides. *Chem. Sci.* **13,** 7429–7436 (2022).
4. Tao, Y. et al. Resonance-Induced Stimuli-Responsive Capacity Modulation of Organic Ultralong Room Temperature Phosphorescence. *J. Am. Chem. Soc.* **144,** 6946−6953 (2022).
5. Xu, Z. et al. Supercooled Liquids with Dynamic Room Temperature Phosphorescence Using Terminal Hydroxyl Engineering. *Angew. Chem. Int. Ed.* **62,** e202301564 (2023).
6. Yang, D.-D. et al. Enhancement of Long-Lived Persistent Room-Temperature Phosphorescence and Anion Exchange with I^- and SCN^- via Metal–Organic Hybrid Formation. *ACS Appl. Mater. Interfaces* **15,** 1495−1504 (2023).
7. Xiao, T. et al. A UV and X-ray dual photochromic Zn (II) metal-organic framework based on viologen: Photo-controlled luminescence and temperature-dependent phosphorescence. *Dyes Pigm.* **208,** 110812 (2022).
8. Yang, D.-D. et al. Multistimuli-Responsive Materials Based on Zn(II)-Viologen Coordination Polymers and Their Applications in Inkless Print and Anticounterfeiting. *Inorg. Chem.* **61,** 7513–7522 (2022).
9. Yang, D.-D. et al. Two multifunctional stimuli-responsive materials with room-temperature phosphorescence and their application in multiple dynamic encryption. *Mater. Chem. Front.* **6,** 2709–2717 (2022).

10. Wei, W.-J., Mu, Y., Wei, L., Hu, J.-X. and Wang, G.-M. Two Photochromic Complexes Assembled by a Nonphotochromic Ligand: Photogenerated Radical Enhanced Room-Temperature Phosphorescence. *Inorg. Chem.* **60,** 108–114 (2021).
11. Feng, D.-X. et al. Photochromic Dy-Phosphonate Assembled by a Pyridine Derivative: Synthesis, Structure, and Light-Enhanced Room-Temperature Phosphorescence. *Cryst. Growth Des*. **22,** 5680–5685 (2022).
12. Di, Y.-M., Li, M.-H., You, M.-H., Zhang, S.-Q. and Lin, M.-J. Photochromic and Room Temperature Phosphorescent Donor–Acceptor Hybrid Crystals Regulated by Core-Substituted Naphthalenediimides. *Inorg. Chem*. **60,** 16233–16240 (2021).
13. Di, Y.-M., Li, M.-H., Zhang, S.-Q., You, M.-H. and Lin, M.-J. Photochromic and Room-Temperature Phosphorescent D–A Hybrid Crystals Induced by Anion−π Interactions. *Cryst. Growth Des.* **21,** 3511−3520 (2021).
14. Feng, D.-X. et al. Light-Induced Electron Transfer Toward On/Off Room Temperature Phosphorescence in Two Photochromic Coordination Polymers. *Adv. Funct. Mater.* **33,** 2305796 (2023).
15. Yang, D.-D. et al. A series of zinc coordination compounds showing persistent luminescence and reversible photochromic properties via charge transfer. *Chem. Eng. J.* **466,** 143202 (2023).
16. Ding, B., Gao, H., Wang, C. and Ma, X. Reversible room-temperature phosphorescence in response to light stimulation based on a photochromic copolymer. *Chem. Commun*. **57,** 3154–3157 (2021).
17. Li, Y., Gu, F., Ding, B., Zou, L. and Ma, X. Photo-controllable room-temperature phosphorescence of organic photochromic polymers based on hexaarylbiimidazole. *Sci. China Chem*. **64,** 1297–1301 (2021).
18. Yao, X. et al. Dynamic room-temperature phosphorescence by reversible transformation of photo-induced free radicals. *Sci. China Chem*. **65,** 1538–1543 (2022).
