# Supplementary material for: Zero-dimensional halide hybrid bulk glass exhibiting reversible photochromic ultralong phosphorescence
Source: Nat Commun. 2024 Jun 29;15:5519. doi: 10.1038/s41467-024-49886-7 (PMC11217438; doi:10.1038/s41467-024-49886-7)
Supplement: Supplementary file 3 — Description of Additional Supplementary Files [file 41467_2024_49886_MOESM3_ESM.pdf]

Description of Additional Supplementary Files:

Supplementary Data 1: Crystallographic data of P-Zn crystal (CCDC number: 2323188).

Supplementary Data 2: Result files from theoretical calculations.

Supplementary Movie 1: Luminescent behavior of P-Zn-BP glass with binary dot arrays under the irradiation of a UV lamp at 365 nm.
